# Prevalence and socio-demographic correlates of the compliance with the physical activity guidelines in children and adolescents in Germany

**DOI:** 10.1186/1471-2458-12-714

**Published:** 2012-08-30

**Authors:** Darko Jekauc, Anne K Reimers, Matthias O Wagner, Alexander Woll

**Affiliations:** 1 Department of Sport Science, University of Konstanz, Constance, 1078457, Germany

**Keywords:** Physical activity guideline, Socio-demographic, Children, Adolescents, Compliance

## Abstract

**Background:**

Regular physical activity (PA) is a prerequisite for normal growth and maturation, health, and fitness of children and adolescents. Because of the growing evidence of the health benefits of regular PA, several national and international public health organisations have established PA guidelines. The purpose of this study was to assess the percentage of children and adolescents in Germany who meet the PA guideline of 60 minutes of moderate-to-vigorous PA each day and to evaluate socio-demographic correlates of compliance with the PA guideline.

**Methods:**

The sample consists of 4,529 children and adolescents aged between 4 and 17 years who lived and were registered in the Federal Republic of Germany between 2003 and 2006. The compliance with the PA guideline was assessed using a widely accepted screening measure. Socioeconomic status, immigration background and residential area were assessed using a parent questionnaire.

**Results:**

Overall, 13.1% of girls and 17.4% of boys complied with the national guideline of 60 minutes of moderate-to-vigorous PA daily. While compliance was significantly lower in older participants of both gender groups, the steepest decrease in compliance was observed for age groups around the transition time from primary to secondary school. Logistic regression revealed that socioeconomic status and a migration background were significant predictors for compliance in girls and residential area for compliance in boys.

**Conclusions:**

Programs and policy action addressing the problem of decreasing compliance with PA guideline with increasing age are warranted. The transition from primary school to secondary school seems to be a critical stage in life with respect to PA behaviour. Therefore, specific interventions should aim at restructuring and reorganising their daily and physical activities during this transition.

## Background

The increase in prevalence of overweight and obesity in children and adolescents is a major public health concern in industrialised countries [1] and physical activity (PA) is inversely related to being overweight and obesity [2]. Especially for children and adolescents, regular PA is a prerequisite for normal growth and maturation, health, and fitness. Compared to inactive young people, physically active children and adolescents have higher levels of cardiorespiratory fitness, muscular endurance and muscular strength, more favourable cardiovascular and metabolic disease risk profiles, enhanced bone health, and reduced symptoms of anxiety and depression [3]. In addition, limited PA during childhood and adolescence may predispose development of a sedentary life style in adulthood [4,5].

Because of the growing evidence of the health benefits of regular PA, several public health organisations have established PA guidelines [6-11]. These organisations claim that children and adolescents aged between 5 and 17 years should perform at least 60 minutes of moderate to vigorous PA (of 5 to 8 metabolic equivalent of task) each day. Data from the HBSC-Study (Health Behaviour in School-aged Children) showed that in most European countries most adolescents aged 11, 13 and 15 years do not engage in moderate-to-vigorous PA every day [12], and similar results were reported for the United States [13], Australia [14] and Brazil [15].

Three comprehensive reviews concluded that socio-demographic variables are consistently correlated with PA in children and adolescents and that boys are more active than girls [16-18]. However, data on age-related levels of PA are inconclusive. For instance, while Sallis et al. [16] reported that younger children were more active than older children, the results by Uijtdewillingen et al. [17] were opposite. In addition, van der Horst et al. [18] did not find enough evidence that would suggest that age is a determinant of PA. Further, being of African-American descent has been associated with lower PA levels [17] and adolescents of Euro-American race are more physically active [16] suggesting that ethnicity influences PA level. Finally, while the review of Sallis et al. [16] concluded that socioeconomic status is not related to PA, Stalsberg and Pedersen [19] reported that 58% of the reviewed articles found that adolescents from families with higher socioeconomic status tend to be more physically active than those from families with lower socioeconomic status.

To date, representative data on comparative prevalence rates and potential predictors for compliance with the PA guideline in German children and adolescents are lacking. Previous studies had several limitations including (i) lack of a representative nationwide study sample, (ii) prevalence estimates only provided for specific and narrow age groups and (iii) use of non-validated measures to estimate compliance with the PA guideline. Hence, statements on compliance with the PA guideline and potential predictors of children and adolescents in Germany can not be generalised, and are incomplete and unreliable.

The purpose of this study was to assess the percentage of children and adolescents in Germany who meet the PA guideline of 60 minutes of moderate-to-vigorous PA each day and to evaluate the socio-demographic correlates of guideline compliance using nationwide representative data, a broad age range and reliable and validated measurement instruments.

## Methods

### Sampling and participants

Data were obtained from the Motorik Modul (MoMo-Study), a study which aims to estimate prevalence rates in PA and level of motor abilities in children and adolescents in Germany [20]. The sample of the MoMo-Study is a representative sub-sample of the German Health Interview and Examination Survey for Children and Adolescents (KiGGS) [21]. To ensure the representativeness of the study results, deviations of the sample from the population structure regarding age, gender, region and country of citizenship oversampling procedures were used. Participants were recruited using a three step process. First, a systematic sample of 167 primary sampling units was selected from an inventory of German communities stratified according to the BIK classification system [21] that measures the level of urbanization and the geographic distribution. The probability of any community being picked was proportional to the number of inhabitants younger than 18 years. For communities with less than 350 inhabitants under 18 years, the adjacent community was added to the sample. Second, an age stratified sample of randomly selected children and adolescents was drawn from the official registers of local residents for the KiGGS with a total of 28,400 participants aged between 0 und 17 years [22]. Out of these 28,400 selected participants, 17,641 children and adolescents aged between 0 and 17 years took part in the KiGGS for a response rate of 62.1%. Third, 7,866 participants aged between 4 and 17 years in the KiGGS-sample were randomly assigned to the sample of the MoMo-Study. Of these 7,866 participants 4,529 children and adolescents took part in the MoMo-Study (response rate = 57.6%). In accordance with this three step recruitment process, weighting procedures were used to ensure the representativity of the results. In the first step, design weights were calculated which are the products of the inverse selection probabilities of each community and each participant within the community according to age and gender. In the second step, adjustment weighting was conducted to adjust the deviations of the design weighted net sample of the KiGGS from the population structure (31.12.2004) considering age, gender, region (East vs. West Germany) and nationality (German vs. not German) [22]. In a third step, inverse selection probabilities were calculated to rule out deviations of the net sample of the MoMo-Study from the KiGGS net sample. A set of variables as age, gender, social status, migration background, residential area, physical activity, motor abilities, subjective and objective measures of health status were used to assess the deviations between the MoMo-Study and the KiGGS and to calculate the selection probabilities [23]. The results of these analyses showed that age, socioeconomic status, migration background and subjective health had a significant effect on participation rates [23]. The final weight was a product of these three steps weightings which was normed to the sample size of the MoMo-Study.

Informed written consent was obtained from the participants and their parents or guardians before the subjects entered into the study according to Helsinki Declaration. The study was approved by the ethics committee of the Charité of the Humboldt University of Berlin in March 2003.

### Data collection

Data were collected at central locations at the 167 sample points rented for the KiGGS and MoMo studies. The MoMo-Study was conducted approximately two weeks after the examination of the KiGGS-Study at the same location. All potential participants of the MoMo-Study were asked by the personnel of the examiner team of the KiGGS for permission to be contacted by the team of the MoMo-Study. In the cases where permission was granted, the potential participants were firstly contacted by letter containing a pamphlet to provide basic information on the MoMo-Study and secondly by telephone to arrange an appointment at the examination location. In order to improve participation rate participants aged 10 years or younger received a small present (e. g. cuddly toy) and participants aged 11 years or older received an incentive of 10 Euro. Data were collected between May 2003 and May 2006 [20]. Participants of the MoMo-Study aged younger than 11 years were interviewed in the presence of their parents by qualified interviewers. Participants aged 11 years or older completed the PA questionnaire independently.

### Measures

#### Physical activity

Compliance with the PA guideline was assessed by two items asking about the engagement in bouts of moderate-to-vigorous PA for at least 60 minutes during the past 7 days and during a typical week [24]. Moderate-to-vigorous PA was defined in the questionnaire as "any activity that increases your heart rate and makes you get out of breath some of the time". The participants were asked to sum the duration of all times spent in moderate-to-vigorous PA for each day across all activities.

For this study, a qualified staff member (native speaker) translated the PA screening measure developed by Prochaska et al. [24] from English to German. Another staff member translated the questionnaire from German back into English without reference to the original instrument. The comparison of the re-translated version with the original version revealed one wording difference that was subsequently resolved by the translators. Finally, the German version of the PA screening measure was completed by five seventh-grade students to test the comprehensibility of the translated measure. These five students did not participate in the main study. The German and English version of the PA screening measure contain the same item and scale formatting.

A separate study was conducted with 196 participants aged between 9 and 17 years to assess reliability and validity of this PA screening measure [25]. The test-retest reliability was sufficient: the composite measure showed a significant intraclass correlation of 0.74 for a one-week test-retest interval. In addition, the PA index significantly correlated with accelerometer measured (moderate-to-vigorous) PA (Actigraph GT1M) (r = 0.24) and with (moderate-to-vigorous) PA measured using the Previous Day Physical Activity Recall (PDPAR) (r = 0.43). These results are comparable with the reliability and validity estimates of the English version of the PA screening measure [24].

#### Socio-demographic characteristics

Socio-demographic characteristics were assessed using a parent questionnaire conducted in the KiGGS [21]. The questionnaire included items on both parents' educational and professional status as well as total income available to the family household [26]. The categorisation of the socioeconomic status was conducted according to Winkler and Stolzenberg [26]. The migration background variable was constructed based on information on nationality, country of birth, year of immigration of both parents as well as historical criteria (e.g. labour migrants, asylum seekers), languages spoken at home and the proficiency of German language of both the children and parents [21]. The residential area variable reflected the level of urbanisation. Towns or settlements with less than 5,000 residents were classified as rural area, towns with 5,000–19,999 residents as small town, towns with 20,000–99,999 residents as medium-sized town and cities with 100,000 or more residents as city.

### Data analysis

Data was analysed using the Statistical Package for Social Science (SPSS) version 19 (IBM, New York, USA). Frequency distributions were calculated for all predictors and for the outcome variable (compliance with the PA guideline). To calculate the PA compliance index, the mean of both PA items was computed. A new dichotomous variable was created differentiating between compliers and non-compliers with the PA guideline. If the participant received a score of seven days with moderate-to-vigorous PA for at least 60 minutes, the participant was assigned to the group of compliers; otherwise the participant was assigned to the group of non-compliers.

Participant’s age, migration background, socioeconomic status and residential area were considered as potential predictors for compliance with the PA guideline. Because of the well-documented differences between boys and girls [16-18], all analyses were calculated separately for both gender groups. All analyses were performed separately for children (4 to 10 years) and adolescents (11 to 17 years) because we expected that the mechanisms of action of the predictors differ between children and adolescents [16]. Bivariate and multivariate analysis strategies were used to rule out confounding effects. Chi-square goodness of fit tests were performed to evaluate bivariate associations between the predictors and the outcome variable. Four multiple logistic regressions were calculated to assess the incremental power of prediction of each predictor among children and adolescents and within each gender group, respectively. Weighting procedures were employed to enhance the representativeness of estimates [22]. To adjust p-values and confidence intervals for clustering effects within the primary sampling units, the SPSS-Module "Complex Samples" was used.

## Results

### Sample characteristics

Frequency distributions for the predictors and the outcome variable are shown in Table 1. The mean age was 11.3 years (SD = 4.1 years). The proportion of boys and girls was almost even. Almost half of the participants had middle socioeconomic status. One in four participants had a low and one in four participants had a high socioeconomic status. Overall, 11.0% of the participants had a migration background. While 58.5% of the participants lived either in a small or a middle-sized town, only 15.6% live in cities with more than 100,000 residents. The average number of days with 60 minutes of moderate to vigorous PA for overall sample was 3.9 days (SD = 2.0 days; boys: 4.2 ± 1.9 days; girls: 3.7 ± 2.0 days). Overall, 15.3% of the participants complied with the PA guideline.

**Table 1:** Frequency distributions for the predictors and the outcome variable

**Variable**	**Total (N = 4529)**	**boys (n = 2285)**	**girls (n = 2244)**
Age group			
4-5 years	989 (21.8%)	484 (21.2%)	505 (22.5%)
6-10 years	1712 (37.8%)	863 (37.8%)	849 (37.8%)
11-13 years	886 (19.6%)	470 (20.6%)	416 (18.5%)
14-17 years	942 (20.8%)	468 (20.5%)	474 (21.1%)
Socioeconomic status			
low	1157 (25.8%)	578 (25.6%)	479 (26.1%)
medium	2195 (49.0%)	1113 (49.3%)	1082 (48.7%)
high	1127 (25.2%)	568 (25.1%)	559 (25.2%)
Migration background			
yes	496 (11.0%)	253 (11.1%)	243 (10.9%)
no	4013 (89.0%)	2025 (88.9%)	1988 (89.1%)
Residential area			
rural area	1173 (25.9%)	593 (26.0%)	580 (25.8%)
small town	1334 (29.5%)	691 (30.2%)	643 (28.7%)
medium-sized town	1315 (29.0%)	645 (28.2%)	670 (29.9%)
city	707 (15.6%)	356 (15.6%)	351 (15.6%)
Compliance with physical activity guideline			
compliers	601 (15.3%)	350 (17.4%)	252 (13.1%)

Note: *N* = total sample size; *n* = group sample size.

### Bivariate analyses

Gender was moderately associated with compliance with the PA guideline: 17.4% of the boys and 13.1% of the girls complied with the PA guideline (*χ*^2^ = 14.0; df = 1; p < .001). Age was a significant predictor for compliance with PA guideline in boys and in girls. The percentage of younger children who met the PA guideline was greater than that of older children (Table 2). Socioeconomic status significantly predicted compliance with the PA guideline only for girls but not for boys. Girls with a low socioeconomic status had the highest prevalence for meeting the PA guideline. Although not significantly, a smaller percentage of girls with migration background (14.2%) complied with the PA guideline than girls without migration background (21.4%).

**Table 2:** Prevalence of the compliance with the physical activity guideline in children

	**boys**				**girls**			
	**compliance**	***χ***^**2**^	**df**	**p**	**compliance**	***χ***^**2**^	**df**	**p**
Age group		12.2	1	.000		12.3	1	.000
4-5 years	35.4%				28.4%			
6-10 years	24.2%				17.9%			
Socioeconomic status		0.5	2	.773		8.1	2	.017
low	25.8%				27.5%			
medium	27.4%				20.0%			
high	28.6%				17.0%			
Migration background		0.1	1	.758		3.2	1	.068
yes	28.4%				14.2%			
no	27.1%				21.4%			
Residential area		7.5	3	.058		2.1	3	.549
rural area	28.9%				19.9%			
small town	27.1%				20.9%			
medium-sized town	22.2%				23.5%			
city	33.0%				18.3%			

Note: *p* = p-value of the chi-square statistic; *df* = degrees of freedom.

In adolescent girls, age, socioeconomic status and migration background were significantly associated with compliance (Table 3). Adolescent girls aged between 11 and 13 years, those with a low socioeconomic status and those with migration background more frequently met the PA guideline. In adolescent boys, socioeconomic status and residential area were significantly associated with PA compliance. The percentage of adolescent boys with a medium socioeconomic status who met the PA guideline was greater than that of those with high or low socioeconomic status. The highest compliance rates with the PA guideline were found in adolescent boys living in rural areas (Table 3).

**Table 3:** Prevalence of the compliance with the physical activity guideline in adolescents

	**boys**				**girls**			
	**compliance**	***χ***^**2**^	**df**	**p**	**compliance**	***χ***^**2**^	**df**	**p**
Age group		0.5	1	.464		4.8	1	.029
11-13 years	9.4%				8.3%			
14-17 years	8.1%				5.0%			
Socioeconomic status		7.1	2	.029		9.1	2	.011
low	7.0%				9.3%			
medium	10.9%				6.7%			
high	5.8%				2.8%			
Migration background		1.9	1	.168		6.7	1	.010
yes	5.4%				10.8%			
no	9.1%				5.5%			
Residential area		20.1	3	.000		1.3	3	.725
rural area	16.5%				7.1%			
small town	7.9%				7.2%			
medium-sized town	6.0%				6.0%			
city	6.6%				5.0%			

Note: *p* = p-value of the chi-square statistic; *df* = degrees of freedom.

### Logistic regression analyses

Table 4 shows the results of the logistic regression analysis to evaluate the predictive power of the correlates of meeting the PA guideline in girls aged between 4 and 10 years. The proportion of explained variance was rather small with Nagelkerke’s R^2^ of .07. The results showed that age, socioeconomic status and migration background significantly influenced the compliance with the PA guideline. The chance of meeting the PA guideline decreased by approximately 17% with every additional year of age. Girls with low socioeconomic status were more than twice as likely to comply with the PA guideline than girls with high socioeconomic status. Similarly, girls without migration background were more than twice as likely to meet the PA guideline than girls with migration background. Residential area did not have a significant effect on compliance with the PA guideline.

**Table 4:** Logistic regression for predicting physical activity guideline compliance in girls aged 4 to 10 years

	**B-Weight**	**SE**	***χ***^**2**^	**df**	**p**	**adj OR**	**95% CI for adj OR**	
							**Lower**	**Upper**
Age (in years)	−0.19	0.04	18.5	1	.000	0.83	0.76	0.90
Socioeconomic status			13.3	2	.001			
low	0.82	0.24	11.3	1	.001	2.28	1.41	3.68
medium	0.20	0.22	0.8	1	.358	1.22	0.80	1.88
high (ref.)	-	-	-	-	-	1.00	-	-
Migration background			5.5	1	.020			
no	0.70	0.30	5.5	1	.020	2.01	1.12	3.60
yes (ref.)	-	-	-	-	-	1.00	-	-
Residential area			1.1	3	.768			
rural area	0.01	0.29	0.0	1	.977	1.01	0.58	1.76
small town	0.08	0.24	0.1	1	.726	1.09	0.68	1.74
medium-sized town	0.22	0.23	0.9	1	.347	1.24	0.79	1.96
city (ref.)	-	-	-	-	-	1.00	-	-
Intercept	−1.00	0.47	4.5	1	.033	0.37		

*N* = 1354; -2 log Likelihood = 858.2; Nagelkerkes R^2^ = .060.

Note: *SE* = standard error; *df* = degrees of freedom; adj *OR* = adjusted odds ratio; *CI* = confidence interval; ref. = reference value.

The results of the logistic regression analysis for boys aged between 4 and 10 years are presented in Table 5. Age and residential area significantly influenced compliance with the PA guideline. The chance of complying with the PA guideline decreased by 15% for every additional year of age. In comparison to boys living in cities, boys residing in middle-sized towns were approximately half as likely to meet the PA guideline.

**Table 5:** Logistic regression for predicting physical activity guideline compliance in boys aged 4 to 10 years

	**B-Weight**	**SE**	***χ***^**2**^	**df**	**p**	**adj OR**	**95% CI for adj OR**	
							**Lower**	**Upper**
Age (in years)	−0.17	004	18.6	1	.000	0.85	0.79	0.91
Socioeconomic status			0.5	2	.772			
low	−0.15	0.22	0.5	1	.486	0.86	0.56	1.31
medium	−0.04	0.18	0.1	1	.815	0.96	0.67	1.37
high (ref.)	-	-	-	-	-	1.00	-	-
Migration background								
no	−0.02	0.23	0.0	1	.943	0.98	0.62	1.55
yes (ref.)	-	-	-	-	-	1.00	-	-
Residential area			7.8	3	.050			
rural area	−0.19	0.23	0.7	1	.418	0.83	0.52	1.31
small town	−0.30	0.21	2.2	1	.140	0.74	0.49	1.10
medium-sized town	−0.57	0.21	7.5	1	.006	0.56	0.38	0.85
city (ref.)	-	-	-	-	-	1.00	-	-
Intercept	0.59	0.38	2.4	1	.119	1.80		

*N* = 1347; -2 log Likelihood = 1053.9; Nagelkerkes R^2^ = .044.

Note: *SE* = standard error; *df* = degrees of freedom; adj *OR* = adjusted odds ratio; *CI* = confidence interval; ref. = reference value.

Similarly to the results for younger girls, age, socioeconomic status and migration background significantly influenced the PA compliance in adolescent girls (Table 6). The chance of meeting the PA guideline decreased by 19% for every additional year of age. Adolescent girls with low socioeconomic status were three times more likely to meet the PA guideline than girls with high socioeconomic status. Contrary to the results for younger girls, female adolescents with migration background were twice as likely to comply with the PA guideline than girls without migration background.

**Table 6:** Logistic regression for predicting physical activity guideline compliance in girls aged 11 to 17 years

	**B-Weight**	**SE**	***χ***^**2**^	**df**	**p**	**adj OR**	**95% CI for adj OR**	
							**Lower**	**Upper**
Age (in years)	−0.21	0.07	9.7	1	.002	0.81	0.71	0.92
Socioeconomic status			6.5	2	.040			
low	1.16	0.46	6.4	1	.011	3.18	1.30	7.75
medium	0.86	0.44	3.8	1	.050	2.36	1.00	5.58
high (ref.)	-	-	-	-	-	1.00	-	-
Migration background			4.8	1	.029			
no	−0.68	0.31	4.8	1	.029	0.51	0.28	0.93
yes (ref.)	-	-	-	-	-	1.00	-	-
Residential area			2.6	3	.453			
rural area	0.60	0.44	1.8	1	.175	1.82	0.77	4.31
small town	0.61	0.40	2.3	1	.130	1.84	0.84	4.06
medium-sized town	0.40	0.40	1.0	1	.328	1.48	0.67	3.28
city (ref.)	-	-	-	-	-	1.00	-	-
Intercept	−0.36	1.08	0.1	1	.741	0.70		

*N* = 890; -2 log Likelihood = 453.6; Nagelkerkes R^2^ = .071.

Note: *SE* = standard error; *df* = degrees of freedom; adj *OR* = adjusted odds ratio; *CI* = confidence interval; ref. = reference value.

In adolescent boys, the only significant predictor for complying with the PA guideline was residential area (Table 7). The chance for meeting the PA guideline was 2.5 times higher for adolescent boys who lived in rural areas than for boys who lived in cities.

**Table 7:** Logistic regression for predicting physical activity guideline compliance in boys aged 11 to 17 years

	**B-Weight**	**SE**	***χ***^**2**^	**df**	**p**	**adj OR**	**95% CI for adj OR**	
							**Lower**	**Upper**
Age (in years)	−0.04	0.06	0.4	1	.502	0.96	0.86	1.07
Socioeconomic status			5.7	2	.057			
low	0.15	0.37	0.2	1	.673	1.17	0.57	2.40
medium	0.61	0.29	4.4	1	.036	1.85	1.04	3.28
high (ref.)	-	-	-	-	-	1.00	-	-
Migration background			0.3	1	.614			
no	0.22	0.43	0.3	1	.614	1.24	0.54	2.88
yes (ref.)	-	-	-	-	-	1.00	-	-
Residential area			15.9	3	.001			
rural area	0.92	0.33	7.7	1	.006	2.51	1.31	4.82
small town	0.09	0.34	0.1	1	.791	1.09	0.56	2.12
medium-sized town	−0.17	0.35	0.2	1	.631	0.84	0.42	1.69
city (ref.)	-	-	-	-	-	1.00	-	-
Intercept	−2.59	0.96	7.3	1	.007	0.07		

*N* = 938; -2 log Likelihood = 604.7 ; Nagelkerkes R^2^ = .048.

Note: *SE* = standard error; *df* = degrees of freedom; adj *OR* = adjusted odds ratio; *CI* = confidence interval; ref. = reference value.

## Discussion

The purposes of this study were to show representative prevalence rates for complying with the PA guideline of 60 minutes of moderate-to-vigorous PA every day in children and adolescents in Germany and to evaluate socio-demographic correlates of compliance with the PA guideline. The results of this study showed that only 15.3% of children and adolescents aged between 4 and 17 years meet the current PA guideline of 60 minutes of moderate-to-vigorous PA every day. These estimates are comparable to results of the HBSC-Study for Germany [12]. The results of both studies emphasise the importance of interventions aimed at enhancing the level of PA in children and adolescents in Germany. The gender differences in compliance with PA guidelines in this study are in agreement with earlier reports for other countries including European countries [12] and the United States [13,27,28]. Both in children and adolescents, boys show higher compliance rate than girls. These differences are especially large in preschool children (4–5 years) where 35.4% of boys and 28.4% of girls meet the PA guideline. For schoolchildren and adolescents, the difference between both gender groups decreases.

A progressive decrease in the prevalence of compliance with the PA guideline with age was observed. Similar age-related decreases have been reported in several international studies with subjective [12] and objective [29] measures of PA. For girls, there is a continuous decrease during childhood and adolescence. In childhood, the chance to meet the PA guideline decreased by 17% every year and during adolescence 19%. Therefore, PA interventions should be employed for girls of all ages. For boys, there was a significant decrease in the prevalence of meeting the PA guideline only during childhood but not during adolescence. Especially, strong decrease in the compliance rate was observed during the transition from childhood to adolescence and hence this stage of life seems to be a particularly critical period for the maintenance of PA levels [30]. In the German school system, most children change from primary school to secondary school around 10 years of age, requiring a reorganisation of everyday activities and friendships. Hence, PA programs need a special focus on this age period.

Interestingly, socioeconomic status influenced compliance with the PA guideline only in girls but not in boys. Contrary to our expectations, girls with low socioeconomic status were more likely to comply with the PA guideline than girls with high socioeconomic status. These differences intensify in adolescence. These results contradict the findings of a previous review [19] that in 58% of the reviewed studies children and adolescents from families with higher socioeconomic status tended to be more physically active than those from families with lower socioeconomic status. We speculate that this discrepancy in results between studies is related to the German school system in which adolescents with higher socioeconomic status more frequently attend a "Gymnasium" (an academic secondary school in the tripartite German secondary school system) and usually spend more time at school especially in the afternoons. Long schooldays, afternoon school and homework presumably pose an organisational challenge for meeting the PA guideline. However, it is unclear why this effect occurs only in girls but not in boys.

Migration background was a predictor of compliance with the PA guideline only for girls but not for boys. Interestingly, coming from a family with migration background had a negative effect on PA compliance in children but a positive effect on PA compliance in adolescents. For girls with migration background, the likelihood of meeting the PA guideline decreased slightly with increasing age. However, for girls without migration background, the compliance with the PA guideline decreased drastically around the transition from childhood to adolescence. Similarly to the socioeconomic effect, we speculate that the German school system may be responsible for these developments. Adolescents without migration background are more likely to attend the "Gymnasium" that is associated with more time spent at school and on homework. However, it remains unclear why the effect of migration background on PA compliance only affects girls but not boys. Further studies are needed to test this assumption and to better understand these gender specific differences.

In contrast to the effects of socioeconomic status and migration background, the effect of residential area significantly influenced compliance with the PA guideline only in boys. We hypothesize that boys who live in rural areas are more likely to use a bicycle and walk longer distances to get to school and to meet with friends. While in cites and medium-sized towns, the public transportation grid is well developed, rural areas suffer from a lack of public transportation. Hence, adolescent boys in rural areas may be forced to be physically active as a means of transportation. One strategy to bring the level of PA up to 60 minutes of moderate-to-vigorous PA per day could be to increase use of active transport by improving the infrastructure (e. g. building safe and convenient bicycle and walking paths) and promoting active transport in schools and communities. Two types of interventions were found to be effective in promoting PA: community-scale and street-scale urban design, as well as land use policies and practices [31]. Urban planners, policy makers and local communities should be involved in such interventions [32]. These endeavours should be implemented especially in such regions (e. g. rural areas) where active transport is safe for children and adolescents. Girls should be encouraged to use this kind of transportation in particular [33].

This work examined the socio-demographic predictors of compliance with the PA guideline. However, one important issue is to question the appropriateness of the PA guidelines. PA guidelines were developed to quantify the amount of PA which is needed to support the normal growth and maturation, health, and fitness of children and adolescents. After reviewing 850 articles on the relationship between PA and health in children and adolescents, Strong et al. [3] conclude that “school-age youth should participate daily in 60 minutes or more of moderate to vigorous physical activity”. Several international [6-11] organisations have adopted this PA recommendation for children and adolescents. However, these strictly formulated PA recommendations raise the question whether the daily regularity of 60 minutes of moderate to vigorous PA is the most important aspect for health benefits or the accumulation of moderate to vigorous PA within one week. Convincing empirical evidence for the daily regularity of PA has not been yet shown. Because this issue has a serious impact on formulating future PA guidelines further research in this field is required. Especially, longitudinal studies are needed to evaluate the merit of PA recommendations.

The major strength of this study is that the subjective measure of compliance with the PA guideline used in this study is a well established instrument [16,25]. In addition, this study reports national level representative data for children and adolescents in Germany for the full age spectrum between 4 and 17 years. However, the results of this study should be interpreted with caution because of some limitations. First, this study has a cross-sectional design, and hence the results of the study do not allow causal inferences of predictors. Second, the results were based on self-report data which has been shown to overestimate the prevalence rates of PA compliance compared to those assessed using objective measures [34]. In fact, compliance rates measured with objective measures may be even lower than those reported in this study. Third, this study did not provide information on the school obligations and transportation grid that would allow for a better understanding of the effects of socio-demographic variables.

## Conclusions

The results of this study showed that a majority of children and adolescents in Germany do not meet the guideline of 60 minutes of moderate-to-vigorous PA every day. Consequently, there is a need for program and policy action as early as possible at the family, community, school, health care, and governmental levels to tackle the problem of decreasing PA with increasing age. The transition from primary school to secondary school appears to be a critical stage of a children’s life with respect to their PA behaviour. Therefore, specific interventions are needed at this stage of life to help young people restructure their daily activities and reimplement PA into daily routines. Especially, boys living in medium seized towns and cities as well as girls with higher socioeconomic status and without migration background were the highest risk groups and need support to develop and maintain habits for daily PA.

## Competing interests

The authors declare that they have no competing interests.

## Authors’ contributions

DJ was responsible for the overall conception and design of this manuscript, statistical analysis, interpretation of data and contributed to the design of the study. AKR provided edits to the paper. MOW contributed to the design of the study and provided edits to the paper. AW contributed to the design of the study. All authors read and approved the final manuscript.

## Pre-publication history

The pre-publication history for this paper can be accessed here:

http://www.biomedcentral.com/1471-2458/12/714/prepub
